# Parent-couple satisfaction, parent depression, and child mental health in families with autistic children

**DOI:** 10.3389/fpsyt.2023.1306456

**Published:** 2024-01-11

**Authors:** Brianna Piro-Gambetti, Jessica Greenlee, Daniel Bolt, Lauren M. Papp, Sigan L. Hartley

**Affiliations:** ^1^Waisman Center, University of Wisconsin-Madison, Madison, WI, United States; ^2^School of Human Ecology, University of Wisconsin-Madison, Madison, WI, United States; ^3^School of Psychology, Lafayette College, Easton, PA, United States; ^4^School of Educational Psychology, University of Wisconsin-Madison, Madison, WI, United States

**Keywords:** autism, autistic, couple, marital, parent, marital satisfaction, mental health, depression

## Abstract

**Introduction:**

Within two-parent households, the parent-couple subsystem (marital or romantic partner relationship) is posited to shape the mental health of both parents and children. Autistic children and their parents have an elevated-risk for mental health problems. The present study longitudinally examined the mediating role of the quality of the parent-couple relationship in time-ordered pathways between changes in the mental health problems of autistic children and in parent depression symptoms at a within-family level.

**Methodology:**

Using four time points of data collected on 188 families of autistic children (aged 5–12 years) across 3 years, the bidirectional associations between parent-couple relationship satisfaction, parent depressive symptoms, and child internalizing and externalizing mental health problems were investigated. Two multi-group (grouped by parent gender) complete longitudinal mediation models in structural equation modeling using Mplus software were conducted.

**Results:**

Parent-couple relationship satisfaction mediated: (1) the association between higher parent depressive symptoms and higher child internalizing mental health problems 12 months later for both mothers and fathers, and (2) the association between higher child externalizing mental health problems and higher father depression symptoms 12 months later. Father depression symptoms mediated a pathway from lower parent-couple satisfaction to higher child internalizing mental health problems 12 months later, and mother depression symptoms mediated the pathway from higher child externalizing mental health problems to lower parent-couple satisfaction 12 months later.

**Conclusion:**

Findings highlight the bidirectional and complex ways that parent and child mental health and the quality of the parent-couple relationship are entwined across time in families of autistic children. Family-wide interventions that address the needs of multiple family members and family systems are best suited to improve the mental health of parents and autistic children.

## 1 Introduction

The Family Systems framework (1) theorizes that the quality of the parent-couple relationship (i.e., marital or romantic partner relationship) is intertwined with the mental health of both parents (2, 3) and children (4, 5). Parents of autistic children are, on average, at risk for short-term and unsatisfying parent-couple relationships relative to other parents (6–9). Making up this average, however, is a wide range of experiences, including many parents of autistic children reporting highly satisfying and long-lasting couple relationships (10). To-date, there are no published longitudinal studies on the role of the parent-couple relationship, a modifiable intervention target, for shaping the mental health of parents and their autistic child. Yet, identifying interventions for improving the health and quality of life of autistic children and their families is of high public health importance given that 1 in 36 children in the U.S. meet criteria for autistic spectrum disorder (11). To address this research gap, the present study examined the bidirectional and time-ordered connections between parent-couple relationship satisfaction, parent depression symptoms, and mental health problems of autistic children across 3 years.

The family system is comprised of both individual family members and unique subsystems that continuously influence one another (1, 12–15). The parent-couple subsystem is often depicted at the center of the family system in two-parent households [e.g., (16, 17)]. Research supports the bidirectional connections between the quality of the parent-couple relationship and parent and child mental health and suggest that the parent-couple relationship may be a mediating conduit through which parent and child mental health influence one another. Indeed, higher parent-couple relationship dissatisfaction has been linked to higher parent depressive symptoms both cross-sectionally and 3 years later (2), and a satisfying parent-couple relationship is thought to buffer parents from child-related stressors by partners serving as a source of emotional support (2, 18). In a bi-directional manner, parent mental health also impacts their couple relationship satisfaction. For example, one’s own and one’s partner’s level of depression and anxiety negatively predicts their parent-couple relationship satisfaction [e.g., (3, 19)]. A depressed parent often withdraws from their partner, becomes more irritable and hostile to their partner, and engages in destructive strategies which foster couple conflict (20, 21). The mental health of women in particular (versus men) has been reported to shape couple interactions [e.g., (3, 22, 23)]. In contrast, an unsatisfying couple relationship has been shown to take a toll on the mental health of men (24), perhaps because men often report that their partner is their primary source of emotional support.

Parent-couple relationship satisfaction also has important associations with child mental health in general population samples [e.g., (4, 25)]. Children exposed to maladaptive parent-couple behaviors (e.g., destructive parent-couple conflict) or otherwise perceive that their parents are unhappy in their parent-couple relationship are at risk for internalizing mental health problems (e.g., feeling anxious and emotionally insecure) (4, 17, 20, 21, 26). For example, a recent study found that if mothers had higher parent-couple relationship satisfaction, their child perceived their family as functioning better, and in turn, had fewer depression symptoms (4).

In the opposite direction, there is evidence that the parenting stressors, including from child mental health problems [e.g., (5, 27, 28)], contribute to decreases in parent-couple relationship satisfaction e.g., (29–31). Indeed, there is evidence from families of neurotypical children that difficulties related to parenting a child with mental health problems, and particularly externalizing mental health problems (e.g., aggressive and disruptive behaviors), often contribute to feelings of parenting stress, fatigue, and depressed affect [e.g., (32)]. In turn, parents may have limited capacity to interact with their partner in an engaged and affectionate manner [e.g., (33)]. Parent-couple relationship dissatisfaction that arises from this tension has been linked to parent depression [e.g., (34)].

Autism spectrum disorder is a lifelong neurodevelopmental disorder characterized by differences related to speech and nonverbal communication, social interactions, and repetitive and restricted behaviors that interfere with everyday functioning (35). Autism is also associated with a plethora of co-occurring mental health problems including both internalizing (i.e., anxious or depressed mood) and externalizing (i.e., disruptive and aggressive behavior) problems (36–38). Indeed, 14–20% of autistic children experience at least one depressive episode before the age of 18 years (39), 40% endure clinically elevated anxiety symptoms (40), 63% have co-occurring attention deficit hyperactivity disorder (41), and approximately 25% are reported to exhibit an aggressive behavior problem (42). Other common co-occurring challenges include oppositional defiant disorder and conduct disorder (43). Parents of autistic children also face a higher risk for clinical depression and depression symptoms compared to parents of children without a developmental disability (44), with mothers of autistic children often reporting more severe depression symptoms than fathers (45).

Parents of autistic children are at risk for negative parent-couple outcomes, including lower parent-couple relationship satisfaction (7, 9), perceptions of decreased partner support and affection (46), and higher rates of divorce or separation (6, 47) when compared to parents of children without developmental disabilities. Moreover, parents of autistic children with more mental health problems report lower parent-couple relationship satisfaction (48, 49) and fewer daily positive parent-couple interactions (50) than do parents of autistic children with fewer mental health problems. Moreover, higher parent-couple conflict and/or lower parent-couple relationship satisfaction predicts increased depression symptoms in mothers of autistic children [e.g., (51)]. Recent evidence also suggests that autistic children respond more negatively (i.e., exhibit more maladaptive emotional, behavioral, and physiological responses) to parent-couple conflict than neurotypical children (52), and may be especially sensitive to the negative impacts of maladaptive parent-couple relationships. However, to date, there are no published longitudinal studies examining the role of the parent-couple relationship as mediator, or conduit, for within-family connections between parent depression and mental health problems of autistic children.

The goal of the current study was to understand whether parent-couple relationship satisfaction explains, in part, the bidirectional time-ordered relations between parent depression and mental health problems in autistic children across four data collection time points, each spaced 12 months apart. A total of 188 mothers and fathers (within-couples) of autistic children independently completed questionnaires assessing their own and their child’s mental health, as well as their level of parent-couple relationship satisfaction. There were two study aims: (1) examine the association between parent-couple relationship satisfaction and parent depression and child mental health problems across 3 years; and (2) determine the extent to which parent-couple relationship satisfaction mediated associations between parent depression symptoms and child mental health problems.

Drawing from research on non-autistic samples, and the Family Systems framework, we hypothesized that: (1) parent depression would predict decreased parent-couple relationship satisfaction 12 months later; (2) child mental health problems would also predict decreased parent-couple relationship satisfaction 12 months later; (3) parent-couple relationship satisfaction would significantly mediate the association between parent depression and child mental health problems across time. Given the evidence linking child mental health to parent mental health in non-autistic populations [e.g., (4, 32, 33)], we also hypothesized that higher child externalizing (vs. internalizing) mental health problems were hypothesized to lead to increases in parent depression symptoms and decreases in parent-couple relationship satisfaction. In contrast, higher parent depression symptoms and lower parent-couple relationship satisfaction were expected to lead to increases in child internalizing mental health problems. Primary hypothesized pathways were: (a) higher parent depression at T1 → decreased parent-couple relationship satisfaction at T2 → increased child mental health problems at T3; and (b) higher child mental health problems at T1 → decreased parent-couple relationship satisfaction at T2 → increased parent depression at T3. Similar pathways are hypothesized from T2-T3-T4. Given prior reports of higher depression symptoms in mothers (versus fathers) of autistic children (45), and evidence of altered time-ordered direction of effects between partner mental health and couple relationship quality in general population samples [e.g., (3)], the above pathways were tested separately in mothers and fathers.

## 2 Materials and methods

The current study used data from T1-T4 of the Family Outcomes in Autism Spectrum Disorder Study (R01MH199091; Hartley). IRB approval was obtained through the University of Wisconsin-Madison, and all parents provided informed consent before participating. At T1, 188 parent-couples participated in the study. Inclusion criteria included: (1) being a parent of child diagnosed with ASD between the age of 5–12 years; (2) part of a committed parent-couple relationship (i.e., defined as in a committed partner relationship 3 + yrs., currently cohabiting with the partner); (3) both parents in the couple had to agree to participate; (4) both parents had to be at least 21 years of age. Recruitment methods included research registries, information distributed within autism clinics, and fliers placed throughout the community and schools. If there was more than one autistic child in the family, the oldest child was the target child and reported on for study purposes. The autistic child had to have a medical or educational diagnosis of ASD and the diagnostic assessment must have included the autistic diagnostic observation schedule [ADOS-2nd edition (53)]. The child’s current level of autism symptoms was measured through parent-report of the Social Responsiveness Scale-2nd Edition [SRS-2 (54)]. The SRS total t-score needed to be greater than 60 to participate in the present study. Five autistic children did not meet this threshold (received a t-score at or below 60), but a review of medical and educational records as well as ADOS scores revealed that these children did indeed meet criteria for ASD. For further demographic information about the families, see Table 1.

**Table 1:** Family sociodemographics.

Demographic	*M* (*SD*)
Mother (*n* = 188)	
Age in years (*M* [*SD*])	38.69 (5.62)
Race/ethnicity (*N* [%])	
White, non-Hispanic	170 (90)
Other	18 (10)
Couple Satisfaction Index (*M* [*SD*])	115.23 (31.43)
Father (*n* = 188)	
Age in years (*M* [*SD*])	40.76 (6.19)
Race/ethnicity (N [%])	
White, non-Hispanic	166 (88)
Other	22 (12)
Couple Satisfaction Index (*M* [*SD*])	117.34 (27.49)
Parent couple	
Couple relationship length, years (*M* [*SD*])	14.55 (5.59)
Household income (*N* [%])	
Less than $20,000	2 (1)
$20,000-$39,999	13 (7)
$40,000 and greater	166 (88)
Target child (*n* =188)	
Male (*N* [%])	162 (86)
Age in years (*M* [*SD*])	7.88 (2.24)
Age of diagnosis (*M* [*SD*], months)	48.17 (22.38)
ID (N [%])	65 (34)
SRS (*M* [*SD*])	77.03 (10.29)
Therapy received	
Occupational therapy (*N* [%])	117 (62)
Physical therapy (*N* [%])	36 (19)
Speech therapy (*N* [%])	137 (73)
Behavioral training and management (*N* [%])	105 (56)

*M*, mean; *SD*, standard deviation; N, sample size; ID, intellectual disability; SRS, Social Responsiveness Scale-2nd edition (54); CBCL, Child Behavior Checklist (55, 56).

### 2.1 Procedure

Parents participated in a 2.5-h study visit that took place either at their home or in a research lab at each time point, spaced approximately 12 months apart. Parents jointly answered sociodemographic questions and then independently reported on parent depression symptoms, child mental health problems, and level of parent-couple relationship satisfaction. Each parent was paid $50 for completing this portion of the study.

### 2.2 Measures

#### 2.2.1 Family sociodemographics

Together, parent-couples answered questions regarding family sociodemographics. Parent information included: (A) parent identified gender (mothers = 1, fathers = 2); (B) parent age (years); (C) household income in US $ (1 = $1–$9,999 to 14 = $160,000+). Additionally, parents also reported on child biological sex (female = 1, male = 2), child age (years), and child presence or absence of an intellectual disability (ID) as determined through either IQ testing or a formal ID diagnosis (0 = no ID, 1 = ID).

#### 2.2.2 Parent depression symptoms

The 20-item Center for Epidemiological Studies-Depression Scale [CES-D (57)] was separately completed by parents. Each item was rated on a 4-point scale with 0 indicating rarely or none of the time to 3 indicating most or all of the time. Example items from the CES-D include “I thought my life had been a failure” and “I felt that everything I did was an effort.” A total score greater than or equal to 16 indicates clinically significant depression symptoms (57). The CES-D revealed high internal consistency in mothers (Cronbach’s α = 0.92–0.93) and fathers (Cronbach’s α = 0.89–0.93) across T1-T4. For the means, standard deviations, and t-values for mother- and father-reported CES-D total scores across time, see Table 2.

**Table 2:** Mother and father reported means, standard deviations, and *t*-values for main variables.

Measure	Mother *M*^1^(*SD*)^2^	Father *M*(*SD*)	*t*-value^3^	*df* ^4^	value of *p*
Time 1	*n* = 188	*n* = 188			
CES-D Total^5^	13.49 (10.33)	11.52 (8.90)	2.289	184	0.023^*^
CBCL Int. T^6^	62.99 (9.55)	61.84 (9.66)	1.527	187	0.128
CBCL Ext. T^7^	60.05 (11.12)	59.65 (10.30)	0.629	187	0.530
CSI Total^8^	115.23 (31.43)	117.34 (27.49)	−1.034	186	0.303
Time 2	*n* = 162	*n* = 156			
CES-D Total	18.40 (7.10)	16.44 (6.40)	3.029	154	0.003^**^
CBCL Int. T	61.37 (9.14)	60.66 (9.21)	1.103	156	0.272
CBCL Ext. T	57.62 (9.96)	57.84 (9.90)	−0.468	156	0.640
CSI Total	114.13 (35.18)	117.51 (28.67)	−1.470	152	0.144
Time 3	*n* = 138	*n* = 133			
CES-D Total	14.86 (11.29)	11.14 (9.51)	3.103	130	0.002^**^
CBCL Int. T	61.42 (9.17)	59.37 (9.44)	2.317	131	0.022^*^
CBCL Ext. T	56.96 (10.10)	56.93 (10.28)	0.419	131	0.676
CSI Total	115.78 (35.61)	118.26 (29.67)	−0.693	128	0.489
Time 4	*n* = 125	*n* = 122			
CES-D Total	14.68 (10.70)	12.40 (10.41)	2.199	116	0.030^*^
CBCL Int. T	61.07 (9.30)	60.48 (9.05)	0.741	120	0.460
CBCL Ext. T	56.38 (10.56)	56.54 (10.98)	−0.073	120	0.942
CSI Total	117.61 (31.64)	117.53 (29.11)	0.049	113	0.961

^1^mean; ^2^standard deviation; ^3^value for paired-samples *t*-test; ^4^degrees of freedom; ^5^Center for Epidemiological Studies-Depression Scale total score (57); ^6^Child Behavior Checklist Internalizing Problems T-Score (56); ^7^Child Behavior Checklist Externalizing T-Score (56); ^8^Couple Satisfaction Index total score (58); ^*^p < 0.05; ^**^ *p* < 0.01.

#### 2.2.3 Child mental health problems

The Child Behavior Checklist [CBCL (55, 56)] preschool form (ages 1.5–5 years) and school age form (ages 6–18 years) were utilized in order to assess child mental health problems. Parents complete this 113-item questionnaire by separately rating each item on a 3-point scale (0 = not true to 2 = very or often true). The current study utilized the internalizing and externalizing t-score in model analyses. The CBCL internalizing scale consists of 32 items and is separated into three subscales: (1) anxious/depressed; (2) withdrawn/depressed; (3) somatic complaints. Example items include, “Feels worthless or inferior,” “There is very little he/she enjoys,” and “Overtired without good reason.” The CBCL externalizing scale is composed of 35 items and is broken into two categories: (1) rule-breaking behavior; (2) aggressive behavior. Example items include, “Does not seem to feel guilty after misbehaving,” “Destroys things belonging to his/her family or others,” and “Sudden changes in mood or feelings.” The CBCL is highly reliable within the ASD population (59) and had a high internal consistency across T1-T4 for both mothers (internalizing: Cronbach’s α = 0.84 to 0.85; externalizing: Cronbach’s α = 0.90–0.92) and fathers (internalizing: Cronbach’s α = 0.82–0.86; externalizing: Cronbach’s α = 0.89–0.90) in the present study. Table 2 provides means, standard deviations, and *t*- values for mother- and father-reports of the CBCL internalizing and externalizing t-scores across time.

#### 2.2.4 Parent-couple relationship satisfaction

The Couple Satisfaction Index [CSI (58)] assessed parent-couple relationship satisfaction. This 32-item questionnaire is broken into a series of 6-point scales, with higher numbers representing greater parent-couple relationship satisfaction. An example item is, “In general, how often do you think that things between you and your partner are going well? [0 = never to 5 = all of the time]. A score of 104.5 or below indicates relationship dissatisfaction. For the present study, the number of mothers scoring below the CSI cutoff of 104.5 was as follows: (T1) *n* = 59; (T2) *n* = 56; (T3) *n* = 45; (T4) *n* = 30. The number of fathers scoring below the CSI cutoff was: (T1) *n* = 62; (T2) *n* = 44; (T3) *n* = 33; (T4) *n* = 30. The CSI had high internal consistency in the present study for both mothers (Cronbach’s α = 0.98–0.99) and fathers (Cronbach’s α = 0.97–0.98) across T1-T4 and has been used in previous research for parents of autistic children (60).

### 2.3 Data analysis plan

Boxplots and descriptive statistics were used to understand the distribution of the data. An attrition analysis was used to determine if families who completed all time points differed from families with missing data on at least 1 time point. A series of bivariate Pearson correlations allowed us to examine the associations among the main study variables and with family sociodemographics. Family sociodemographics significantly associated with one or more of the independent or dependent variables at two or more time points were included as covariates in primary analyses. Specifically, the independent and dependent variables were regressed on the relevant significant family sociodemographic variables and the unstandardized residual scores were saved and entered in the structural equation model (SEM).

**Figure 1:**
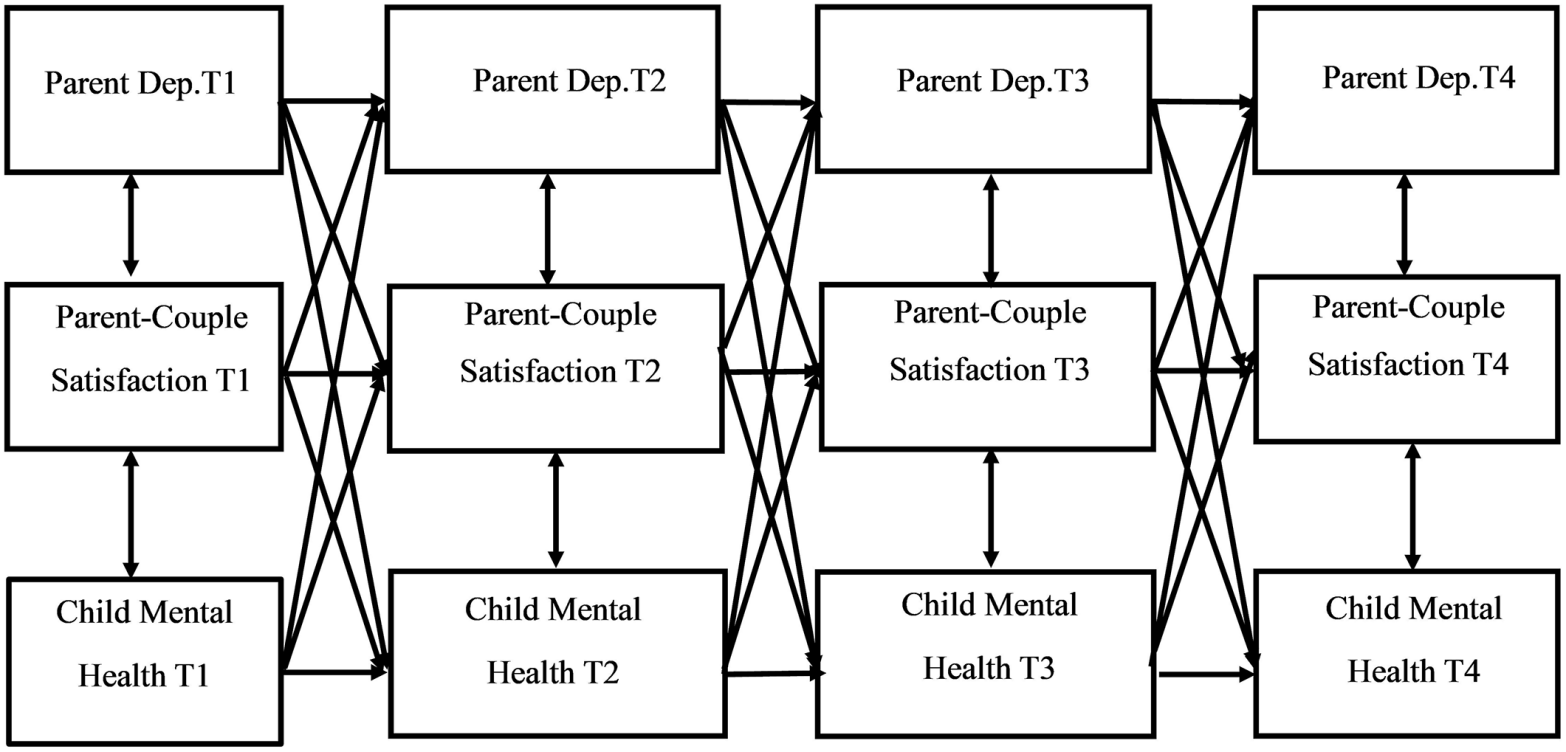
Conceptual model of complete longitudinal mediation model for the associations between parent depression, parent-couple relationship satisfaction, and child mental health problems.

The primary analytic model was a multi-group complete longitudinal mediation model conducted in SEM using Mplus statistical software (61); the recommended software for this type of mediation model (62). Data was from T1-T4 of the Family Outcomes in ASD study. For a conceptual model, see Figure 1. Due to previous research in non-autistic samples suggesting possible differences between mother reactions versus father reactions within the parent-couple relationship [e.g., (21, 22, 45)] our model was grouped by parent gender. This approach provided separate results for mother- and father reported measures and investigated the impact of parent-couple satisfaction on the parent and child mental health connection. The recommended 10:1 ratio of cases/observations to estimated parameters (63, 64) suggests that a minimum total sample size of 360 is needed to detect a meaningful effect, with a minimum of 100 mothers and 100 fathers for each group. By using a complete longitudinal mediation model rather than a focused model, we are able to analyze multiple potential longitudinal associations. In other words, the complete model provides for the examination of a multitude of potential mediational pathways, allowing us to explore both parent and child-driven pathways across the data collection time points (62). Bias-corrected bootstrapped confidence intervals based on 5,000 iterations were included, aligning with best practices for evaluating indirect effects (65). Examining confidence intervals for the indirect effects allowed us to determine if significant mediations exist within the model. The Tucker-Lewis Index (TLI), comparative fit index (CFI), and the root mean square error of approximation (RMSEA) were also examined in addition to the chi-square (*χ*2) test, to evaluate global model fit. A good model fit includes CFI and TLI values greater than 0.90 and an RMSEA value between 0.05 and 0.08 (66, 67). Missing data was accounted for via the full information maximum likelihood method, a robust estimator in SEM (67, 68).

## 3 Results

### 3.1 Preliminary analyses

Assessments of normality revealed that parent depression and child mental health problems were approximately normally distributed (kurtosis range for CES-D = 0.978–2.467; CBCL internalizing = −0.212–2.321; CBCL externalizing = −0.550–0.096). Missing completely at random (MCAR) tests suggested that MCAR was reasonable (*X*^2^ = 6.449, *p* > 0.05). At T1, 188 families provided data from both parents (*N* = 376 parents). Of these families, at T2, data from both parents was available from 155 families. Seven additional families provided mother-report only, and 1 family provided father-report only, for a total of 163 families (*N* = 318 parents). Data from both parents was available from 131 families at T3. Seven additional families provided mother-report only, and 2 families provided father-report only, for a total of 140 families (*N* = 271 parents). At T4, data from both parents was available for 117 families. Eight additional families provided mother-report only, and 5 additional families provided father-report only for a total of 130 families (*N* = 247 parents). Participants lost to attrition indicated moving or not having enough time at the present study cycle to participate as main reasons for leaving the study. Table 1 provides sociodemographic information for the sample.

Attrition analyses were conducted to determine whether families who completed the study at all four cycles (coded 1 for “completers”) differed from families who had missing data at one or more study cycles (coded 2 for “non-completers”). Independent *t*-tests indicated that reports of parent depression (ts: −1.388–0.177, *p* > 0.05) and child mental health problems (internalizing ts: −0.883–0.976, *p* > 0.05; externalizing ts: −1.442–0.422, *p* > 0.05) did not differ significantly for “completers” versus “non-completers.” At T1 (t(373) = 2.266, *p* = 0.012) and T4 (t(227) = 1.789, *p* = 0.037), “completers” reported greater parent-couple satisfaction than “non-completers..” Additionally, parent age at T1 was significantly different for “completers” versus “non-completers” (t(372) = −2.089, *p* = 0.019), with “completers” being slightly younger (*M* = 39.19, *SD* = 5.60) than “non-completers” (*M* = 40.51, *SD* = 6.48). There were no significant differences between the two groups for household income.

Paired-sample t-tests indicated that mother- and father-reports of child internalizing mental health problems were statistically different at T3 (t (131) = 2.317, *p* = 0.022), with mothers reporting higher levels of child internalizing problems than fathers. There were no differences between mother- and father-report of child externalizing mental health problems (ts: −0.468–0.629, *p* > 0.05). Mothers and fathers did, however, report statistically different parent depression scores (T1: t(184) = 2.289, *p* = 0.023; T2: t(154) = 3.029, *p* = 0.003; T3: t(130) = 3.103, *p* = 0.002; T4: t(116) = 2.199, *p* = 0.030), with mothers reporting higher levels of depression symptoms than fathers at each time point. Mothers and fathers did not report significantly different parent-couple satisfaction scores (ts: −1.470 to 0.049, *p* > 0.05). Means, standard deviations, and within-couple differences between mother- and father-reports are provided in Table 2.

Results from bivariate Pearson correlations among main study variables and sociodemographics are in Table 3. Parent depression and child mental health problems were significantly positively associated for both mother- (internalizing model rs: 0.191–0.416, *p* < 0.05; externalizing model rs: 0.158–0.419, *p* < 0.05) and father-reports (internalizing model rs: 0.198–0.436, *p* < 0.05; externalizing model rs: 0.202–0.371, *p* < 0.05). Parent-couple satisfaction was significantly negatively associated with child mental health problems for both mother-(internalizing model rs: −0.278 – −0.176, *p* < 0.05; externalizing model rs: −0.301 – −0.157, *p* < 0.05) and father-reports (internalizing model rs: −0.460 – −0.177, *p* < 0.05; externalizing model rs: −0.311 – −0.169, *p* < 0.05). Similarly, parent-couple satisfaction was significantly negatively correlated with parent depression symptoms for mother-report (rs: −0.444 – −0.218, *p* < 0.05) and father-report (rs: −0.522 – −0.250, *p* < 0.05).

**Table 3:** Correlations between main study variables and sociodemographics.

	1	2	3	4	5	6	7	8	9	10	11	12	13	14	15	16	17	18	19	20	21
1.PAge^1^	–	−0.014	0.076	0.048	0.080	0.059	−0.065	0.021	−0.150	−0.086	−0.017	−0.085	0.046	0.077	0.088	0.002	−0.015	0.121	0.121	0.137	0.065
2.Income	−0.045	–	0.044	0.063	−0.148^*^	−0.136	.-172^*^	−0.067	−0.044	0.074	0.059	0.028	0.075	0.058	0.080	0.089	0.121	−0.013	−0.047	−0.065	−0.018
3.CAge^2^	−0.007	0.101	–	0.012	−0.062	−0.037	−0.100	−0.047	−0.010	0.050	0.114	0.164	0.063	0.020	0.188*	0.089	0.099	−0.131	−0.095	−0.152	−0.074
4.Sex^3^	−0.006	0.082	0.012	–	−0.086	−0.073	−0.052	−0.051	0.018	0.021	−0.056	0.035	0.065	−0.018	−0.007	−0.037	−0.048	0.099	0.059	0.012	0.105
5. ID^4^	0.024	−0.148^*^	−0.062	−0.086	–	0.024	0.043	−0.053	0.063	−0.031	−0.023	−0.087	−0.091	−0.032	−0.055	−0.045	−0.074	0.111	0.099	0.085	0.100
6. Dep1^5^	0.050	−0.164^*^	−0.024	0.065	0.009	–	0.553^**^	0.699^**^	0.704^**^	−0.392^**^	−0.380^**^	−0.367^**^	−0.221^*^	0.287^**^	0.318^**^	0.396^**^	0.389^**^	0.277^**^	0.326^**^	0.371^**^	0.349^**^
7. Dep2	0.063	−0.170^*^	0.003	−0.019	−0.024	0.462^**^	–	0.589^**^	0.691^**^	−0.238^**^	−0.396^**^	−0.351^**^	−0.282^**^	0.191^*^	0.224^**^	0.245^**^	0.246^**^	0.158^*^	0.185^*^	0.218^*^	0.141
8. Dep3	0.076	−0.180^*^	−0.073	0.070	−0.010	0.691^**^	0.524^**^	–	0.717^**^	−0.357^**^	−0.358^**^	−0.444^**^	−0.313^**^	0.308^**^	0.317^**^	0.413^**^	0.348^**^	0.372^**^	0.360^**^	0.419^**^	0.311^**^
9. Dep4	0.041	−0.252^**^	0.073	−0.081	−0.130	0.671^**^	0.429^**^	0.736^**^	–	−0.237^**^	−0.282^**^	−0.218^*^	−0.278^**^	0.232^**^	0.354^**^	0.440^**^	0.416^**^	0.241^**^	0.320^**^	0.303^**^	0.309^**^
10. CSI1^6^	0.059	0.004	0.020	−0.052	−0.001	−0.344^**^	−0.098	−0.336^**^	−0.267^**^	–	0.798^**^	0.774^**^	0.758^**^	−0.040	−0.084	−0.102	−0.175	−0.089	−0.157^*^	−0.257^**^	−0.220^*^
11. CSI2	−0.055	0.074	0.086	−0.052	−0.012	−0.330^**^	−0.382^**^	−0.522^**^	−0.366^**^	0.745^**^	–	0.789^**^	0.800^**^	−0.134	−0.189^*^	−0.140	−0.278^**^	−0.185^*^	−0.264^**^	−0.201^*^	−0.183^*^
12.CSI3	−0.028	0.057	0.097	−0.062	−0.053	−0.319^**^	−0.250^**^	−0.487^**^	−0.436^**^	0.741^**^	0.794^**^	–	0.875^**^	−0.068	−0.148	−0.176^*^	−0.248^**^	−0.124	−0.225^*^	−0.301^**^	−0.247^**^
13. CSI4	0.068	0.014	0.089	−0.013	−0.005	−0.312^**^	−0.159	−0.495^**^	−0.459^**^	0.764^**^	0.745^**^	0.854^**^	–	−0.018	−0.070	−0.108	−0.118	−0.143	−0.204^*^	−0.288^**^	−0.236^*^
14. Int.1^7^	0.147^*^	0.000	−0.010	0.127	−0.017	0.296^**^	0.143	0.229^**^	0.209^*^	−0.130	−0.177^*^	−0.134	−0.184	–	0.661^**^	0.637^**^	0.631^**^	0.622^**^	0.445^**^	0.379^**^	0.391^**^
15. Int.2	0.127	−0.041	0.123	0.116	0.047	0.315^**^	0.133	0.242^**^	0.251^**^	−0.156	−0.236^**^	−0.239^**^	−0.156	0.590^**^	–	0.704^**^	0.717^**^	0.435^**^	0.506^**^	0.368^**^	0.381^**^
16. Int.3	0.026	−0.017	0.046	0.063	−0.032	0.289^**^	0.164	0.275^**^	0.207^*^	−0.259^**^	−0.309^**^	−0.156	−0.460^**^	0.362^**^	0.532^**^	–	0.785^**^	0.401^**^	0.404^**^	0.536^**^	0.442^**^
17. Int.4	−0.021	0.092	0.090	0.114	−0.010	0.436^**^	0.198^*^	0.316^**^	0.338^**^	−0.307^**^	−0.343^**^	−0.329^**^	−0.253^**^	0.569^**^	0.670^**^	0.535^**^	–	0.390^**^	0.445^**^	0.407^**^	0.496^**^
18. Ext.1^8^	0.162^*^	−0.084	−0.140	0.072	0.155^*^	0.308^**^	0.113	0.290^**^	0.241^**^	−0.169^*^	−0.241^**^	−0.282^**^	−0.261^**^	0.607^**^	0.446^**^	0.268^**^	0.456^**^	–	0.761^**^	0.679^**^	0.737^**^
19. Ext.2	0.152	−0.094	−0.155	0.132	0.112	0.288^**^	0.143	0.239^**^	0.202^*^	−0.138	−0.204^*^	−0.190^*^	−0.128	0.411^**^	0.562^**^	0.298^**^	0.486^**^	0.747^**^	–	0.767^**^	0.749^**^
20. Ext.3	0.209^*^	−0.121	−0.226^*^	0.079	−0.020	0.364^**^	0.165	0.371^**^	0.265^**^	−0.270^**^	−0.273^**^	−0.229^**^	−0.311^**^	0.335^**^	0.362^**^	0.435^**^	0.427^**^	0.699^**^	0.789^**^	–	0.840^**^
21. Ext.4	0.102	−0.047	−0.135	0.066	0.147	0.346^**^	0.129	0.275^**^	0.243^**^	−0.199^*^	−0.158	−0.205^*^	−0.214^*^	0.421^**^	0.436^**^	0.281^**^	0.584^**^	0.746^**^	0.805^**^	0.856^**^	–

Pearson Correlations. Mother-report is shaded and above the diagonal. Father-report is unshaded and below the diagonal. ^1^parent age; ^2^child age; ^3^child biological sex; ^4^child intellectual disability; ^5^Center for Epidemiological Studies-Depression Scale (57); ^6^Couple Satisfaction Index total score (58); ^7^Child Behavior Checklist Internalizing Problems T-Score (56); ^8^Child Behavior Checklist Externalizing Problems T-Score (56); ^*^ = *p* < 0.05; ^**^ = *p* < 0.01.

Parent age was associated with father-report of child mental health problems (T1 internalizing: *r* = 0.147*, p* = 0.046; T1 externalizing: *r* = 0.162, *p* = 0.027; T3 externalizing: *r* = 0.209, *p* = 0.016). Household income was significantly associated with both mother-report (T2: *r* = −0.172, *p* = 0.032) and father-report (T1: *r* = −0.164, *p* = 0.028; T2: *r* = −0.170, *p* = 0.036; T3: *r* = −0.180 *p* = 0.041; T4: *r* = −0.252, *p* = 0.006) of parent depression. Child biological sex was not significantly associated with any of the main study variables (*p* > 0.05). Child age was associated with mother-report of child internalizing mental health problems at T2 (*r* = 0.188, *p* = 0.016) and father-report of child externalizing mental health problems at T3 (*r* = −0.226, *p* = 0.009). Child ID status was associated with father-report of child externalizing mental health problems (T1 externalizing: *r* = 0.155, *p* = 0.033). The complete-longitudinal mediation models controlled for parent age and household income. We regressed the main study variables (parent depression, child internalizing mental health problems, child externalizing mental health problems, and parent-couple relationship satisfaction) on parent age and household income at each time point, and the unstandardized residual scores were entered into the mediation models.

### 3.2 Complete-longitudinal mediation models

**Figure 2:**
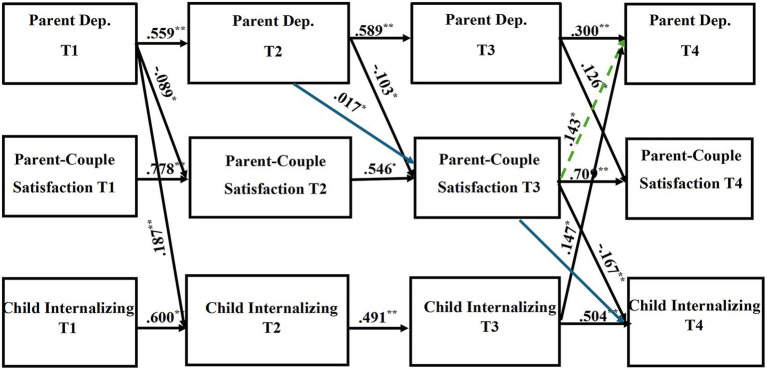
Results of the complete longitudinal mediation model for *mother-report* of parent depression symptoms, parent-couple relationship satisfaction, and child *internalizing* mental health problems, controlling for parent age and household income (Nonsignificant paths removed). Values are standardized path estimates. Blue lines indicate a significant *indirect* path. The green dotted line indicates loss of significance after secondary analyses were conducted. Lagged paths are excluded from figure for simplicity. ^*^p < 0.05; ^**^p < 0.01.

**Figure 3:**
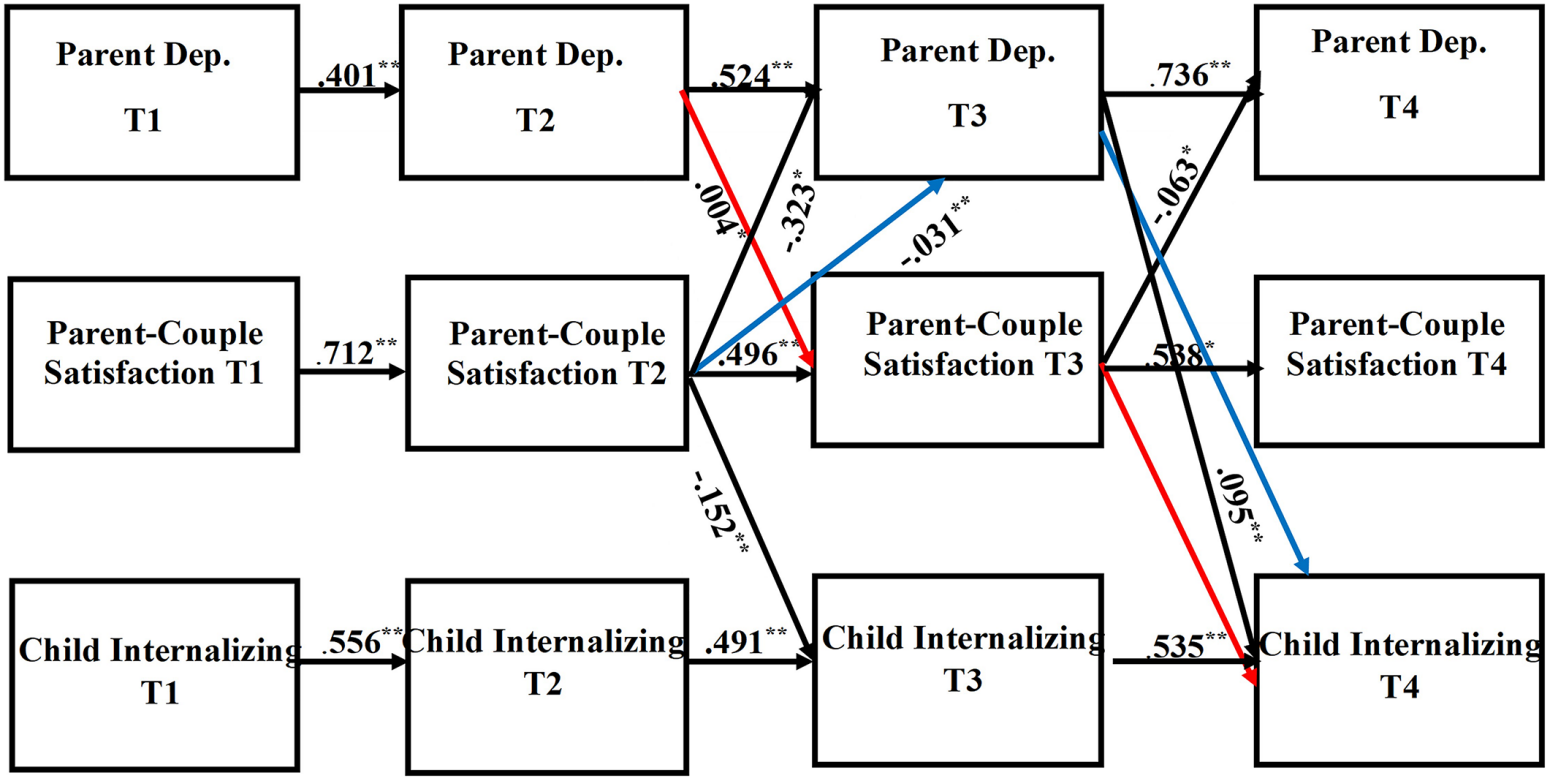
Results of the complete longitudinal mediation model for *father-report* of parent depression symptoms, parent-couple relationship satisfaction, and child *internalizing* mental health problems, controlling for parent age and household income *(Nonsignificant paths removed).* Values are standardized path estimates. Red and blue lines indicate significant *indirect* pathways. Lagged paths are excluded from figure for simplicity. ^*^p < 0.05; ^**^p < 0.01.

**Figure 4:**
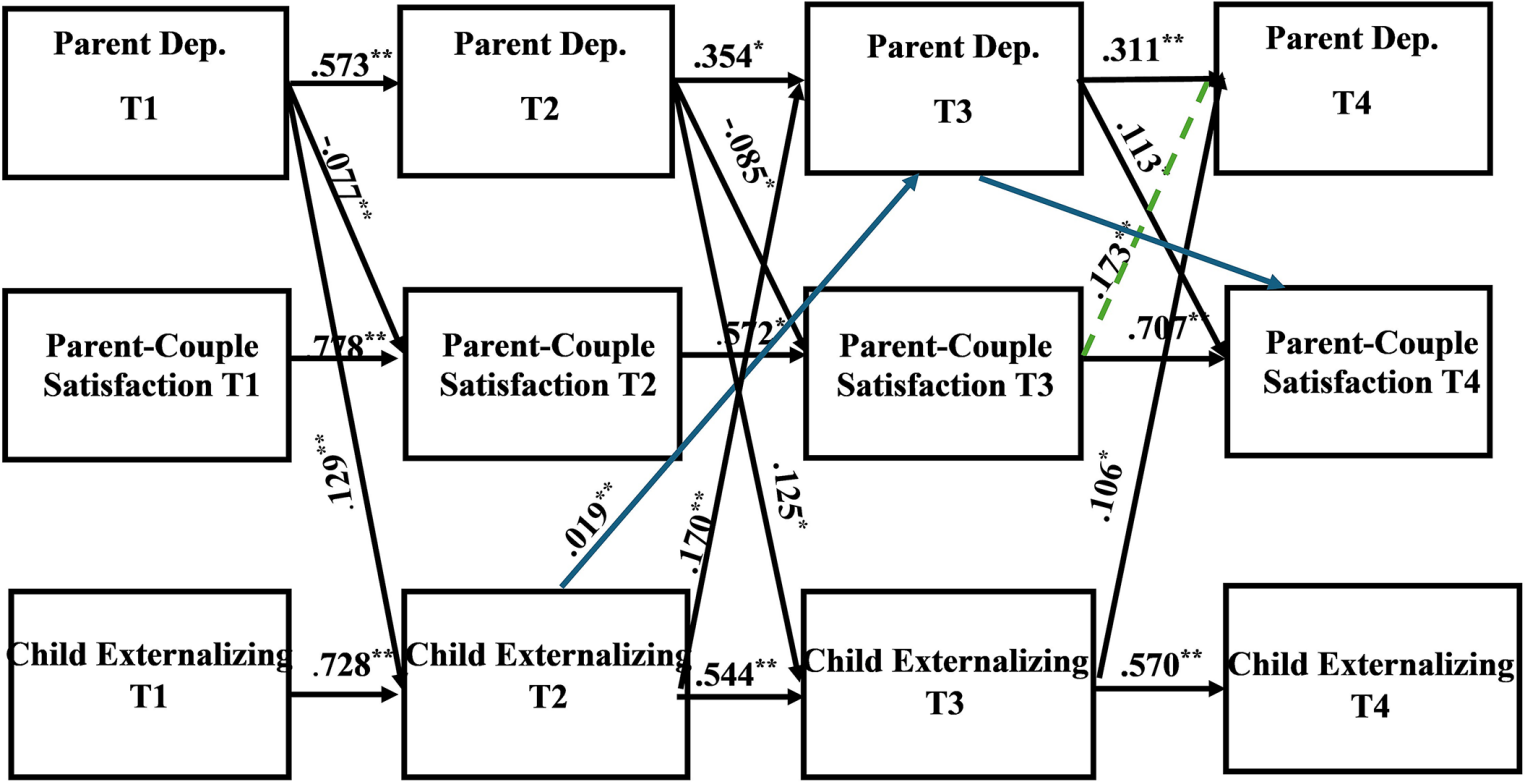
Results of the complete longitudinal mediation model for *mother-report* of parent depression symptoms, parent-couple relationship satisfaction, and child *externalizing* mental health problems, controlling for parent age and household income *(Nonsignificant paths removed).* Values are standardized path estimates. Blue lines indicate a significant *indirect* path. The green dotted line indicates loss of significance after secondary analyses were conducted. Lagged paths are excluded from figure for simplicity. ^*^p < 0.05; ^**^p < 0.01.

**Figure 5:**
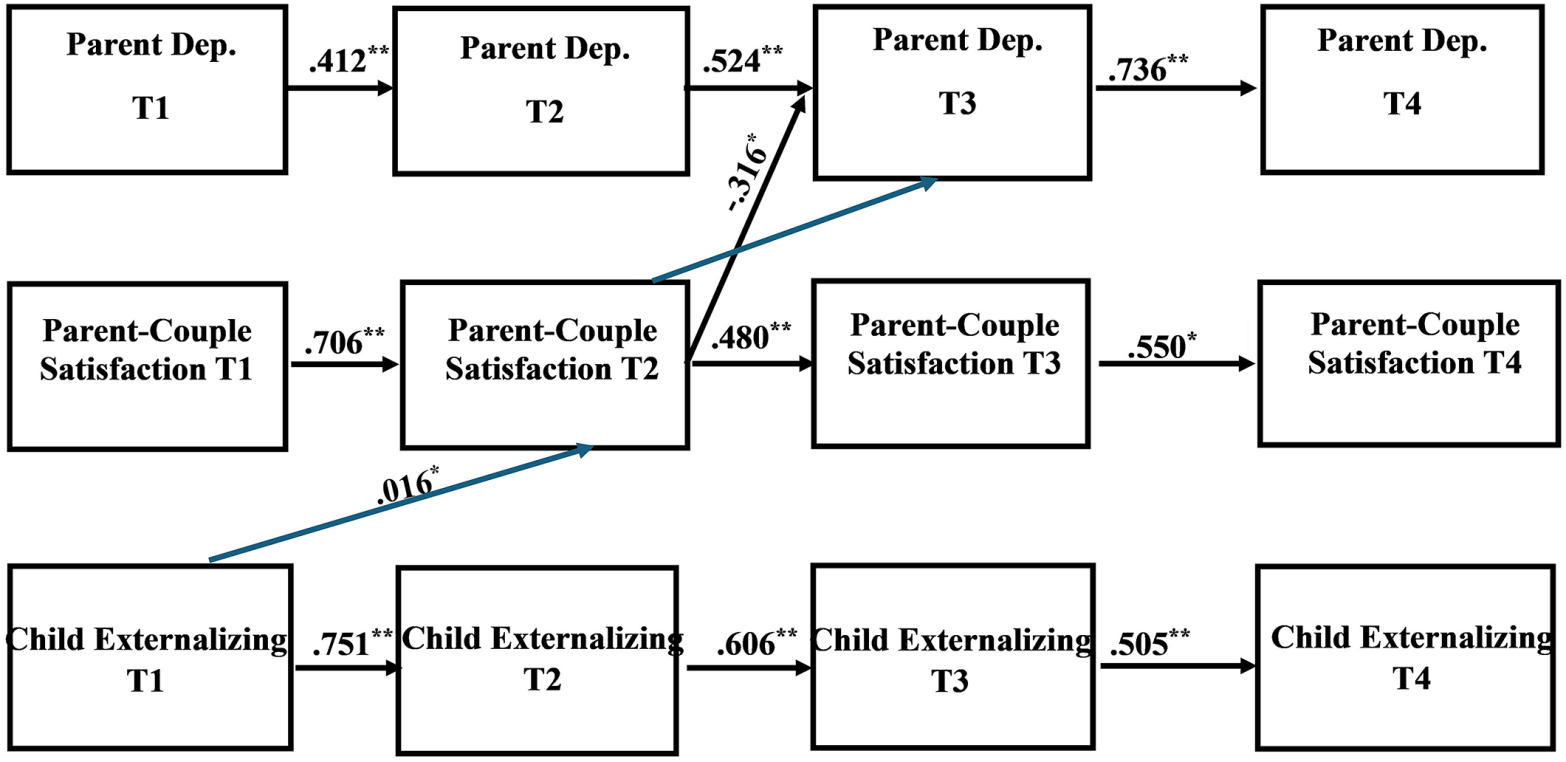
Results of the complete longitudinal mediation model for *father-report* of parent depression symptoms, parent-couple relationship satisfaction, and child *externalizing* mental health problems, controlling for parent age and household income *(Nonsignificant paths removed).* Values are standardized path estimates. Blue lines indicate a significant *indirect* path. Lagged paths are excluded from figure for simplicity. ^*^p < 0.05; ^**^p < 0.01.

Path coefficients of the direct and indirect pathways for the child internalizing mental health problems model can be found in Tables 4, 5, respectively. Tables 6, 7 provide the direct and indirect pathway coefficients for the child externalizing mental health problems model. Figure 1 provides a conceptual model illustrating all possible effects that were analyzed. Figures 2, 3 illustrate the complete-longitudinal mediation models for exploring mother- and father-reported child internalizing mental health problems, respectively. Figures 4, 5 depict the complete-longitudinal mediation models for mother- and father-reported child externalizing mental health problems, respectively.

**Table 4:** Path coefficients for mother- and father-reports of parent depression, parent-couple relationship satisfaction, and child internalizing mental health problems.

Time point	Mother-report *β^1^*(*SE^2^*), unstandardized	Mother-report *β*(*SE*), standardized	Father-report *β*(*SE*), unstandardized	Father-report *β*(*SE*), standardized
Cross effects	CES-D^3^ ➔ CSI^4^			
1➔2	−0.310(0.083)^**^	−0.089(0.029)^*^	−0.251(0.449)	−0.075(0.124)
2➔3	−0.522(0.195)^*^	−0.103(0.041)^*^	−0.206(0.436)	−0.042(0.080)
3➔4	0.351 (0.135)^*^	0.126(0.046)^*^	−0.282(0.426)	−0.090(0.130)
	CES-D ➔ CBCL Int.^5^			
1➔2	0.167(0.060)^*^	0.187(0.052)^**^	0.133(0.101)	0.122(0.082)
2➔3	0.166(0.143)	0.124(0.127)	0.044(0.113)	0.028(0.079)
3➔4	−0.011(0.028)	−0.013(0.037)	0.095(0.022)^**^	0.095(0.019)^**^
	CSI ➔ CES-D			
1➔2	−0.004(0.008)	−0.017(0.039)	0.015(0.040)	0.066(0.160)
2➔3	−0.019(0.036)	−0.060(0.118)	−0.105(0.038)^*^	−0.323(0.096)^*^
3➔4	0.041(0.019)^*^	0.143(0.055)^*^	−0.021(0.012)	−0.063(0.032)^*^
	CSI ➔ CBCL Int.			
1➔2	0.011(0.025)	0.038(0.086)	−0.008(0.030)	−0.024(0.085)
2➔3	0.013(0.015)	0.051(0.054)	−0.051(0.012)^**^	−0.152(0.042)^**^
3➔4	−0.044(0.012)^**^	−0.167(0.037)^**^	−0.030(0.030)	−0.097(0.105)
	CBCL Int. ➔ CSI			
1➔2	−0.073(0.195)	^−^0.019(0.051)	−0.102(0.220)	−0.034(0.071)
2➔3	0.068(0.182)	0.017(0.047)	−0.036(0.193)	−0.011(0.064)
3➔4	−0.106(0.245)	−0.031(0.071)	−0.153(0.344)	−0.051(0.117)
	CBCL Int. ➔ CES-D			
1➔2	0.021(0.042)	0.028(0.059)	0.031(0.049)	0.048(0.070)
2➔3	0.095(0.118)	0.075(0.093)	−0.042(0.069)	−0.042(0.073)
3➔4	0.162(0.068)^*^	0.147(0.062)^*^	0.009(0.050)	0.009(0.045)
Lagged effects	CES-D			
1➔3	0.458(0.106)^**^	0.404(0.082)^**^	0.537(0.045)^**^	0.494(0.045)^**^
	CSI			
	0.286(0.167)	0.248(0.142)	0.392(0.089)^**^	0.349(0.106)^*^
	CBCL Int.			
	0.280(0.114)^*^	0.277(0.124)^*^	0.035(0.147)	0.035(0.154)
2➔4	CES-D			
	0.492(0.093)^**^	0.334(0.051)^**^	0.039(0.148)	0.024(0.091)
	CSI			
	0.154(0.135)	0.170(0.158)	0.114(0.096)	0.112(0.094)
	CBCL Int.			
	0.208(0.071)^*^	0.203(0.077)^*^	0.400(0.150)^*^	0.403(0.154)^*^

^1^Beta value; ^2^Standard Error; ^3^Center for Epidemiological Studies-Depression Scale (57); ^4^Couple Satisfaction Index total score (58); ^5^Child Behavior Checklist Internalizing Problems T-Score (56); ^*^p < 0.05; ^**^p < 0.01.

**Table 5:** Estimates of indirect pathways for child internalizing mental health problems model.

Time point	Pathway	Mother-report β^1^-value of indirect effect	SE^2^	Lower bound of 95% CI^3^	Upper bound of 95% CI	Father-report β-value of indirect effect	SE	Lower bound of 95% CI	Upper bound of 95% CI
1➔2➔3									
	CBCL^4^➔CSI^5^➔CES-D^6^	0.001	0.004	−0.002	0.007	0.011	0.021	−0.015	0.043
	CBCL➔CES-D➔CSI	−0.003	0.005	−0.004	0.002	−0.002	0.003	−0.002	0.000
	CSI➔CBCL➔CES-D	0.003	0.010	−0.013	0.005	0.001	0.007	−0.009	0.002
	CSI➔CES-D➔CBCL	−0.002	0.003	−0.002	0.002	0.002	0.005	0.000	0.009
	CES-D➔CSI➔CBCL	−0.005	0.009	−0.014	−0.002	0.011	0.014	0.002	0.031
	CES-D➔CBCL➔CSI	0.003	0.013	−0.006	0.007	−0.001	0.010	−0.013	0.008
2➔3➔4									
	CBCL➔CSI➔CES-D	0.002	0.005	−0.001	0.004	0.001	0.007	−0.004	0.014
	CBCL➔CES-D➔CSI	0.009	0.010	−0.004	0.022	0.004	0.015	−0.017	0.025
	CSI➔CBCL➔CES-D	0.008	0.010	0.000	0.009	−0.001	0.008	−0.013	0.000
	CSI➔CES-D➔CBCL	0.001	0.003	−0.004	0.001	**−0.031** ^**^	0.010	−0.039	−0.021
	CES-D➔CSI➔CBCL	**0.017** ^*^	0.008	0.006	0.025	0.004^*^	0.002	−0.001	0.003
	CES-D➔CBCL➔CSI	−0.004	0.013	−0.026	0.000	−0.001	0.019	−0.041	0.000

Estimates are standardized values. ^1^Beta-value; ^2^Standard Error; ^3^Bias-Corrected Bootstrap Confidence Interval; ^4^Child Behavior Checklist Internalizing Sub-scale (56);^5^Couple Satisfaction Index (58); ^6^Center for Epidemiological Studies-Depression Scale (57); ^*^p < 0.05, ^**^p < 0.01. Significant values are bold.

**Table 6:** Path coefficients for mother- and father-reports of parent depression, parent-couple relationship satisfaction, and child externalizing mental health problems.

Time point	Mother-report *β*^1^(*SE*^2^), unstandardized	Mother-report *β*(*SE*), standardized	Father-report *β*(*SE*), unstandardized	Father-report *β*(*SE*), standardized
Cross effects	CES-D^3^ ➔ CSI^4^			
1➔2	−0.268(0.061)^**^	−0.077(0.021)^**^	−0.245(0.351)	−0.074(0.098)
2➔3	−0.426(0.190)^*^	−0.085(0.037)^*^	−0.203(0.375)	−0.042(0.070)
3➔4	0.313(0.148)^*^	0.113(0.053)^*^	−0.328(0.337)	−0.101(0.101)
	CES-D ➔ CBCL Ext.^5^			
1➔2	0.126(0.030)^**^	0.129(0.037)^**^	0.065(0.046)	0.056(0.045)
2➔3	0.197(0.050)^**^	0.125(0.049)^*^	0.052(0.056)	0.033(0.039)
3➔4	−0.039(0.021)	−0.043(0.025)	0.009(0.047)	0.008(0.039)
	CSI ➔ CES-D			
1➔2	−0.003(0.010)	−0.014(0.047)	0.015(0.041)	0.067(0.163)
2➔3	−0.012(0.035)	−0.036(0.115)	−0.102(0.040)^*^	−0.316(0.106)^*^
3➔4	0.051(0.017)^*^	0.173(0.038)^**^	−0.023(0.018)	−0.069(0.052)
	CSI ➔ CBCL Ext.			
1➔2	−0.012(0.015)	−0.038(0.047)	0.028(0.022)	0.075(0.060)
2➔3	0.006(0.012)	0.020(0.038)	−0.021(0.016)	−0.063(0.052)
3➔4	−0.009(0.012)	−0.029(0.038)	0.013(0.027)	0.035(0.071)
	CBCL Ext. ➔ CSI			
1➔2	−0.216(0.194)	−0.067(0.060)	−0.140(0.093)	−0.050(0.031)
2➔3	−0.139(0.181)	−0.040(0.051)	−0.006(0.191)	−0.002(0.068)
3➔4	−0.093(0.107)	−0.032(0.036)	0.095(0.142)	0.031(0.048)
	CBCL Ext. ➔ CES-D			
1➔2	−0.007(0.042)	−0.010(0.069)	0.006(0.042)	0.010(0.068)
2➔3	0.197(0.058)^*^	0.170(0.042)^**^	0.045(0.062)	0.049(0.063)
3➔4	0.100(0.025)^**^	0.106(0.035)^*^	−0.069(0.095)	−0.068(0.091)
Lagged effects	CES-D			
1➔3	0.449(0.126)^**^	0.394(0.096)^**^	0.475(0.057)^**^	0.442(0.070)^**^
	CSI			
	0.241(0.220)	0.212(0.196)	0.413(0.098)^**^	0.366(0.109)^*^
	CBCL Ext.			
	0.244(0.106)^*^	0.245(0.115)^*^	0.187(0.073)^*^	0.197(0.079)^*^
2➔4	CES-D			
	0.500(0.087)^**^	0.339(0.046)^**^	0.029(0.154)	0.018(0.094)
	CSI			
	0.161(0.127)	0.178(0.152)	0.144(0.065)^*^	0.137(0.060)^*^
	CBCL Ext.			
	0.123(0.184)	0.116(0.176)	0.323(0.152)^*^	0.296(0.127)^*^

^1^Beta value; ^2^Standard Error; ^3^Center for Epidemiological Studies-Depression Scale (57); ^4^Couple Satisfaction Index total score (58); ^5^Child Behavior Checklist Externalizing Problems T-Score (56); ^*^p < 0.05; ^**^p < 0.01.

**Table 7:** Estimates of indirect pathways for child externalizing mental health problems model.

Time point	Pathway	Mother-report β^1^-value of indirect effect	SE^2^	Lower bound of 95% CI^3^	Upper bound of 95% CI	Father-report β-value of Indirect effect	SE	Lower bound of 95% CI	Upper bound of 95% CI
1➔2➔3									
	CBCL^4^➔CSI^5^➔CES-D^6^	0.002	0.013	−0.010	0.011	**0.016** ^*^	0.006	0.015	0.017
	CBCL➔CES-D➔CSI	0.001	0.004	−0.001	0.005	0.000	0.005	−0.001	0.000
	CSI➔CBCL➔CES-D	−0.006	0.004	−0.007	−0.005	0.004	0.009	0.005	0.006
	CSI➔CES-D➔CBCL	−0.002	0.004	−0.004	−0.002	0.002	0.016	−0.001	0.037
	CES-D➔CSI➔CBCL	−0.002	0.005	−0.011	−0.002	0.005	0.004	0.000	0.004
	CES-D➔CBCL➔CSI	−0.005	0.005	−0.011	0.001	0.000	0.005	−0.010	0.003
2➔3➔4									
	CBCL➔CSI➔CES-D	−0.007	0.005	−0.012	0.002	0.000	0.009	−0.004	0.018
	CBCL➔CES-D➔CSI	0.019^**^	0.004	0.009	0.015	−0.005	0.011	−0.009	−0.006
	CSI➔CBCL➔CES-D	0.002	0.002	0.004	0.005	0.004	0.003	−0.002	0.002
	CSI➔CES-D➔CBCL	0.002	0.004	−0.005	0.005	−0.002	0.014	−0.023	0.016
	CES-D➔CSI➔CBCL	0.002	0.003	−0.002	0.005	−0.001	0.004	−0.006	0.000
	CES-D➔CBCL➔CSI	−0.004	0.006	−0.012	0.001	0.001	0.001	0.000	0.001

Estimates are standardized values. ^1^Beta-value; ^2^Standard Error; ^3^Bias-Corrected Bootstrap Confidence Interval; ^4^Child Behavior Checklist Externalizing Sub-scale (56);^5^Couple Satisfaction Index (58); ^6^Center for Epidemiological Studies-Depression Scale (57); ^*^p < 0.05, ^**^p < 0.01. Significant values are bold.

Baseline models for both child internalizing and child externalizing mental health problems testing equality constraints across mothers and fathers were run in order to determine if there was (at a global level) evidence for differences across groups. In each case, we tested nested models in which all regression paths were constrained to be the same across groups versus models in which all paths were allowed to differ. Both the child internalizing and child externalizing models with paths allowed to differ were significantly different from the baseline models (*p* < 0.001), thus indicating significant differences across groups and supporting the separate report of mother and father model coefficients. Additional testing can be applied for individual paths. However, we chose not to report these results, as statistical power for testing such differences is low, especially in cases where variability in the predictors is low for one or both groups.

#### 3.2.1 Internalizing mental health problems model.

The child internalizing mental health problems model indicated good model fit (*X*^2^ (36) = 36.969, *p* = 0.4240; RMSEA = 0.012; TLI = 0.998; CFI = 0.999). Stability effects were present for mother- and father-report of parent depression (Mother: T1-T2 β = 0.559, *p* = 0.000; T2-T3 β = 0.589, *p* = 0.000; T3-T4 β = 0.300, *p* = 0.000; Father: T1-T2 β = 0.401, *p* = 0.000; T2-T3 β = 0.524, *p* = 0.000; T3-T4 β = 0.736, *p* = 0.000) across time. Stability effects were also present for parent-couple relationship satisfaction (Mother: T1-T2 β = 0.778, *p* = 0.000; T2-T3 β = 0.546, *p* = 0.001; T3-T4 β = 0.709, *p* = 0.000; Father: T1-T2 β = 0.712, *p* = 0.000; T2-T3 β = 0.496, *p* = 0.000; T3-T4 β = 0.538, *p* = 0.001) as well as for child internalizing mental health problems (Mother: T1-T2 β = 0.600, *p* = 0.000; T2-T3 β = 0.491, *p* = 0.000; T3-T4 β = 0.504, *p* = 0.000; Father: T1-T2 β = 0.556, *p* = 0.000; T2-T3 β = 0.491, *p* = 0.000; T3-T4 β = 0.535, *p* = 0.000).

Mother-report of parent depression symptoms significantly directly predicted parent-couple relationship satisfaction across the time points (T1 CES-D to T2 CSI: *β* = −0.089, *p* = 0.002; T2 CES-D to T3 CSI: *β* = −0.103, *p* = 0.013; T3 CES-D to T4 CSI: *β* = 0.126, *p* = 0.006). Mother-report of parent depression symptoms at T1 also directly predicted child internalizing mental health problems at T2 (*β* = 0.187, *p* = 0.000). Mother-report of parent-couple relationship satisfaction at T3 directly predicted both parent depression symptoms (*β* = 0.143 *p* = 0.009) and child internalizing mental health problems (*β* = −0.167, *p* = 0.000) at T4. Additionally, Mother-report of child internalizing mental health problems at T3 directly predicted parent depression symptoms at T4 (*β* = 0.147, *p* = 0.017). Mother-report revealed one significant indirect pathway (*β* = 0.017, *p* = 0.025; CI [0.006, 0.025]), indicating that parent depression symptoms at T2 predicted parent-couple relationship satisfaction at T3, which then predicted child internalizing mental health problems at T4. This finding provides support that parent-couple relationship satisfaction may at least partially mediate the association between parent depression and child internalizing mental health problems.

Father-report of parent depression symptoms at T3 directly predicted child internalizing mental health problems at T4 (*β* = 0.095, *p* = 0.000). Father-report of parent-couple relationship satisfaction directly predicted parent depression (T2 CSI to T3 CES-D: *β* = −0.323, *p* = 0.001; T3 CSI to T4 CES-D: *β* = −0.063, *p* = 0.050) as well as child internalizing mental health problems (T2 CSI to T3 CBCL: *β* = −0.152, *p* = 0.000). Two significant indirect pathways were found. First, parent depression symptoms at T2 predicted parent-couple relationship satisfaction at T3, which then predicted child internalizing mental health problems at T4 (*β* = 0.004, *p* = 0.048; CI [−0.001, 0.003]). This pathway suggests that parent-couple relationship satisfaction may serve as at least a partial mediator for the association between parent depression and child internalizing mental health problems. Second, father-report of parent-couple relationship satisfaction at T2 predicted parent depression at T3, which then predicted child internalizing mental health problems at T4 (*β* = −0.031, *p* = 0.002; CI [−0.039, −0.021]), indicating that parent depression partially mediated the association between parent-couple relationship satisfaction and child internalizing mental health problems.

##### 3.2.1.1 Secondary analyses

Mother-report revealed two unexpected findings, which were examined in secondary analyses. There was a significant positive direct effect of T3 parent depression symptoms predicting T4 parent-couple relationship satisfaction (*β* = 0.126, *p* = 0.006). Further examination suggested a suppression effect (69). This statistical phenomenon can occur in longitudinal models that simultaneously include multiple predictors that are correlated. Parent-couple relationship satisfaction (CSI) ratings were highly correlated over time, which caused the β value to switch direction [i.e., from the expected negative to a positive direction; (69)]. Multiple linear regression analysis examining the effects of T1–T3 CSI, T3 CES-D, and T3 CBCL internalizing on T4 CSI indicated that the β- value for T3 CES-D was positive (*β* = 0.302, *p* = 0.054). After removing the highly correlated T1–T3 CSI, however, the β-value for T3 CES-D switched to negative (*β* = −0.885, *p* = 0.002), indicating a suppression effect. When examined in isolation, higher T3 parent depression symptoms predicted a decrease in parent-couple relationship satisfaction at T4. The second unexpected finding was a significant positive direct effect of T3 parent-couple relationship satisfaction predicting T4 parent depression symptoms (*β* = 0.143, *p* = 0.009). Multiple linear regressions were again conducted to examine the effects of T1–T3 CES-D, T3 CSI, and T3 CBCL on T4 CES-D, and findings revealed that the β-value for T3 CSI was positive (*β* =0.038, *p* = 0.042). After removing T1–T3 CES-D from the multiple linear regression, the β-value for T3 CSI switched to negative (*β* = −0.043, *p* = 0.081), but significance did not remain, thus again demonstrating a suppression effect.

#### 3.2.2 Externalizing mental health problems model

The child externalizing mental health problems model also revealed good model fit (*X*^2^ (36) = 45.175, *p* = 0.1405; RMSEA = 0.037; TLI = 0.985; CFI = 0.996). Stability effects were present for parent depression symptoms (Mother: T1-T2 β = 0.573, *p* = 0.000; T2-T3 β = 0.354, *p* = 0.040; T3-T4 β = 0.311, *p* = 0.000; Father: T1-T2 β = 0.412, *p* = 0.000; T2-T3 β = 0.524, *p* = 0.000; T3-T4 β = 0.736, *p* = 0.000), parent-couple relationship satisfaction (Mother: T1-T2 β = 0.778, *p* = 0.000; T2-T3 β = 0.572, *p* = 0.011; T3-T4 β = 0.707, *p* = 0.000; Father: T1-T2 β = 0.706, *p* = 0.000; T2-T3 β = 0.480, *p* = 0.000; T3-T4 β = 0.550, *p* = 0.002), and child internalizing mental health problems (Mother: T1-T2 β = 0.728, *p* = 0.000; T2-T3 β = 0.544, *p* = 0.000; T3-T4 β = 0.570, *p* = 0.000; Father: T1-T2 β = 0.751, *p* = 0.000; T2-T3 β = 0.606, *p* = 0.000; T3-T4 β = 0.505, *p* = 0.000).

Mother-report of parent depression predicted parent-couple relationship satisfaction 12 months later (T1 CES-D to T2 CSI: *β* = −0.077, *p* = 0.000; T2 CES-D to T3 CSI: *β* = −0.085, *p* = 0.021; T3 CES-D to T4 CSI: *β* = 0.113, *p* = 0.031). Parent-depression also predicted child externalizing mental health problems (T1 CES-D to T2 CBCL externalizing: *β* = 0.129, *p* = 0.000; T2 CES-D to T3 CBCL externalizing: *β* = 0.125, *p* = 0.010). Mother-report of parent-couple relationship satisfaction at T3 predicted parent depression at T4 (*β* = 0.173, *p* = 0.000). Additionally, mother-report of child externalizing mental health problems predicted parent depression (T2 CBCL externalizing to T3 CES-D: *β* = 0.170, *p* = 0.000; T3 CBCL externalizing to T4 CES-D: *β* = 0.106, *p* = 0.002). One significant indirect effect was present for mother-report in that child externalizing mental health problems at T2 predicted parent depression at T3, which then predicted parent-couple relationship satisfaction at T4 (*β* = 0.019, *p* = 0.000; CI [0.009, 0.015]) This finding suggests that parent depression may partially mediate the association between child externalizing mental health problems and parent-couple relationship satisfaction.

Father-report revealed one significant direct effect and one significant indirect effect. Directly, father-report of parent-couple relationship satisfaction at T2 predicted parent depression at T3 (*β* = −0.316, *p* = 0.003). Indirectly, father-report of child externalizing mental health problems at T1 predicted parent-couple relationship satisfaction at T2, which then predicted parent depression symptoms at T3 (*β* = 0.016, *p* = 0.007; CI [0.015, 0.017]). This suggests that parent-couple relationship satisfaction may serve as a partial mediator for the association between child externalizing mental health problems and parent depression symptoms.

##### 3.2.2.1 Secondary analyses

The child externalizing mental health problems model also revealed two suppression effects for mother-report. We conducted follow up multiple linear regressions to examine the unexpected positive effect between T3 parent depression and T4 parent-couple relationship satisfaction (*β* = 0.113, *p* = 0.031). The β-value for T3 CES-D was positive (*β* = 0.347, *p* = 0.022) for the original multiple linear regression examining effects for T1–T3 CSI, T3 CES-D, and T3 CBCL externalizing on T4 CSI. After removing T1–T3 CSI, the β-value for T3 CES-D switched to negative (*β* = −0.644, *p* = 0.019), suggesting that higher parent depression at T3 predicted lower parent-couple satisfaction at T4. Similarly, multiple linear regressions were conducted to investigate the positive effect between T3 parent-couple relationship satisfaction and T4 parent depression (*β* = 0.173, *p* = 0.000). The multiple linear regression investigating the effects of T1-T3 CES-D, T3 CSI, and T3 CBCL externalizing revealed a positive β value for T3 CSI (*β* = 0.042, *p* = 0.026). After removing T1–T3 CES-D, the β-value for T3 CSI switched to negative, but was no longer significant (*β* = −0.039, *p* = 0.156). Additionally, because the significant mother-reported indirect effect included a pathway impacted by a suppression effect (T2 CBCL externalizing ➔ T3 CES-D ➔T4 CSI), secondary analyses were warranted. Specifically, we conducted a Sobel Test (70) using SPSS statistical software (Path A: *β* = 0.344, SE = 0.067; Path B: *β* = −1.048, SE = 0.189; Path C: *β* = −0.039, SE = 0.194; Sobel Test: *z* = −3.77, SE = 0.096, *p* = 0.000), which indicated that the indirect pathway was indeed significant and in the expected direction in that greater child externalizing mental health problems predicted greater parent depression, which in turn, predicted lower parent-couple relationship satisfaction.

## 4 Discussion

The quality of the parent-couple relationship influences both parent and child mental health (2, 4). Parents of autistic children are at risk for poor parent-couple relationships, yet little is known about how this relationship is tied to the mental health of parents and autistic children across time. The present study examined whether parent-couple relationship satisfaction mediated, or accounted for, the time-ordered associations between parent depression and child internalizing and externalizing mental health problems for families with autistic children.

We found that mother depression negatively predicted parent-couple relationship satisfaction 12 months later across all time points. In contrast, for fathers, parent-couple relationship satisfaction negatively predicted his depression symptoms 12 months later (internalizing model: T2 to T3, T3 to T4; externalizing model: T2 to T3). These findings align with previous literature showing that parent mental health problems often take a toll on satisfaction with the couple relationship [e.g., (19, 21, 22)]. In a transactional manner, being dissatisfied with one’s parent-couple relationship satisfaction may contribute to depression symptoms e.g., (2, 3). It is unclear why within families of autistic children, mother depression symptoms predicted declines in her parent-couple relationship satisfaction, but the opposite direction of effects was true for fathers (i.e., low parent-couple relationship satisfaction predicted increases in depression symptoms in fathers). In our sample, mothers reported significantly more depression symptoms than fathers, which has also been reported in prior studies (45). This higher severity of depression symptoms may be why these symptoms lead to declines in satisfaction with the parent-couple relationship in mothers but not in fathers. Alternatively, evidence from neurotypical samples suggests that the mental health of wives (versus husbands) is more likely to foster negative couple interactions [e.g., (3, 22, 23)], whereas negative couple relationships are more likely to lead to mental health problems in husbands (24). Thus, it is possible that the low parent-couple relationship satisfaction takes a greater toll on father mental health than mother mental health within families of autistic children. Perhaps this is because men often report relying heavily on their wives for emotional support (71).

In the current study, mother depression symptoms were bidirectionally related to the autistic child’s internalizing and externalizing mental health problems. This aligns with previous research showing important links between the mental health of parents and that of their autistic child [e.g., (52, 72)]. In contrast, in our complete models, father depression symptoms predicted increased internalizing mental health problems in their autistic child, but not vice versa. Previous research has found that mothers of autistic children tend to report higher levels of parenting stress [e.g., (73, 74)], and higher parenting stress due to child mental health problems may lead to increased maternal depression symptoms (72). Research has also found that mothers of autistic children often take on greater childcare responsibilities than fathers (75, 76). Our results may simply suggest that mothers in our sample experience more frequent exposure to their child’s mental health problems than fathers, and the stressors associated with managing a child’s mental health may take a greater toll on their own mental health. Alternatively, perhaps the parent-couple relationship is acting as a buffer from the negative effects of child mental health problems for fathers as is suggested by previous research [e.g., (2, 18)]. If the father is satisfied with their parent-couple relationship, their mental health may not be as negatively impacted by parenting related stressors (e.g., child mental health problems). More research focused on father’s perspectives of parental depression as well as child mental health problems is needed to better understand this connection. Overall, this suggests that autistic children are sensitive to depression in both mothers and fathers and mothers are directly affected by the mental health problems of their autistic children.

In support of our hypothesis, for both mothers and fathers, parent-couple relationship satisfaction mediated (or accounted for) the association between higher parent depression symptoms and increased child internalizing mental health problems. In other words, higher parent depression predicted lower relationship satisfaction a year later, which subsequently predicted higher child internalizing symptoms the following year. Moreover, father’s parent-couple relationship satisfaction mediated an association between higher child externalizing mental health problems and later increases in father depression symptoms. Thus, relative to mothers whose mood appears to be more directly affected by child mental health problems, father’s mood is negatively affected by child mental health problems through an altered parent-couple relationship. These mediation effects, which are supported by Family Systems Theory, show that the parent-couple relationship is an important conduit through which parent depression shapes child mental health problems and vice versa across time. It is important to build empirical support for a family systems approach specific to autistic families given the implications for prevention and intervention efforts. For instance, this study provides initial empirical evidence that incorporating parent mental health and couple relationship support into early autism intervention could have implications for the child’s mental health as they develop. Existing programs should take a broader family-systems perspective to attend to the effects of parents on children, children on parents, as well as the role of the parent-couple relationship.

The mediation pathways for child internalizing mental health problems were parent driven (parent depression ➔ child internalizing; parent-couple relationship satisfaction ➔ child internalizing). This direction of effects aligns with previous general population research suggesting that child exposure to parent depression symptoms or tension/conflict within the parent-couple relationship can promote child internalizing symptoms (i.e., retreating inward, feeling isolated, etc.) [e.g., (4, 17)]. Alternatively, the mediation pathways for child externalizing mental health problems were child driven (child externalizing ➔ parent-couple relationship satisfaction; child externalizing ➔ parent depression). This direction of effects is supported by both general population research [e.g., (32)] as well as ASD research [e.g., (77–79)], proposing that the parenting stressors associated with child externalizing mental health problems can lead to mental health problems for the parent. Overall, this pattern suggests that parent mental health and the parent-couple relationship are more sensitive to outwardly directed and/or disruptive child mental health problems than to inwardly directed child mental health problems. In contrast, a family environment involving high parent depression and/or a dissatisfied parent-couple relationship appears to contribute to internalized behaviors such as anxiety and depression in the autistic child. Understanding differential pathways of effect within the family system can help tailor intervention. If an autistic child is having difficulties with externalizing behaviors, it may be important for practitioners to monitor parent and couple outcomes. In contrast, if parents are dealing with depression or couple instability, practitioners may want to observe the autistic child for co-occurring internalizing problems. Of course, both parent and child mental health do not exist in a vacuum, and it is important for all members of the family system that individuals receive assistance for mental health problems as needed. But the current findings suggest that a “broad strokes” approach may not be effective for autistic children and their families compared to more tailored services.

In summary, the parent-couple relationship is an important conduit that links parent and child mental health across time within families of autistic children. In addition, mother’s mood appears to have direct ties with the mental health problems of their autistic child whereas father’s mood may be most sensitive to changes in the parent-couple relationship that result from child mental health problems. Thus, there are multi-directional feedback loops connecting parent depression, parent-couple relationship quality, and the mental health problems of autistic children within families.

## 5 Strengths, limitations, and future directions

The current study had several strengths. It leveraged rich longitudinal data, that included both mother- and father- report, and examined multiple parent- and child- driven pathways simultaneously through the utilization of a complete longitudinal mediation model. The use of structural equation modeling allowed us to investigate our complex hypotheses and better understand the family system from multiple directions. Models also separately examined child internalizing and externalizing mental health problems, which were found to have different patterns of association with parent depression and the parent–child relationship.

There were also study limitations. The sample consisted primarily of White, non-Hispanic families with a mid-level socioeconomic status and focused on mother–father couples. More diverse samples are needed to reflect varied family experiences. Additionally, parents who completed all four study cycles were younger and reported greater parent-couple relationship satisfaction, on average, than parents who did not complete one or more study cycles. While there were no significant differences between “completers” and “non-completers,” on reports of parent depression or child mental health problems, it is possible that parents who were more dissatisfied with their parent-couple relationship did not have the motivation to engage in research, particularly in a study in which they both participate and answer questions about their relationship. Additionally, due to the age range of autistic children in the present study (5–12 years at T1), we utilized t-scores from both the CBCL preschool and school-aged forms rather than the raw scores, which may have provided conservative results. It would be interesting to examine associations using CBCL raw scores in future research. Further, given our sample size and the longitudinal nature of our study, we were limited in statistical power to test parallel mediation models of child internalizing and externalizing mental health problems simultaneously; however, our separate examination of internalizing and externalizing problems can guide future larger-sized studies in their effort to determine which is more predictive of outcomes or more impacted by predictors. Moreover, parents experiencing depression symptoms may be biased in their reporting of parent-couple relationship satisfaction and child mental health problems. Observational data capturing actual parent-couple interactions may help reduce this bias and provide a clearer picture of relationship satisfaction. Finally, while the present study asked whether the autistic children received various treatments (e.g., occupational therapy, behavioral training), we did not ask specific information such as the quality of services or the hours received for each service. Future research should consider more detailed information regarding treatment/intervention services.

## 6 Study implications

The parent-couple relationship plays a key role in shaping the family environment and in parent and child mental health. Findings from the current study illuminate the need for family-wide interventions that can help enhance the parent-couple relationship while also targeting parent and child mental health. For example, interventions that combine techniques aimed at improving mental health, such as cognitive behavioral therapy or mindfulness, with couple counseling strategies such as those in Emotionally Focused Therapy (80) or the Gottman Method (81) that help couples identify maladaptive patterns in their interactions and build effective communication may be able improve the overall family environment.

## Data availability statement

The raw data supporting the conclusions of this article will be made available by the authors, without undue reservation.

## Ethics Statement

The studies involving humans were approved by University of Wisconsin-Madison IRB. The studies were conducted in accordance with the local legislation and institutional requirements. The participants provided their written informed consent to participate in this study.

## Author contributions

BP-G: Conceptualization, Formal analysis, Writing – original draft. JG: Writing – review & editing. DB: Conceptualization, Writing – review & editing. LP: Writing – review & editing. SH: Conceptualization, Funding acquisition, Supervision, Writing – review & editing.
